# Calcineurin inhibitors stimulate Kir4.1/Kir5.1 of the distal convoluted tubule to increase NaCl cotransporter

**DOI:** 10.1172/jci.insight.165987

**Published:** 2023-04-10

**Authors:** Dan-Dan Zhang, Xin-Peng Duan, Kerim Mutig, Franziska Rausch, Yu Xiao, Jun-Ya Zheng, Dao-Hong Lin, Wen-Hui Wang

**Affiliations:** 1 Department of Physiology, College of Basic Medical Sciences, Jilin University, Changchun, China.; 2 Department of Pharmacology, New York Medical College, Valhalla, New York, USA.; 3 Institute of Translational Physiology, Charité – Universitätsmedizin, Berlin, Germany.; 4 Department of Pharmacology, Institute of Pharmacy, I.M. Sechenov First Moscow State Medical University, Moscow, Russia.; 5 Department of Physiology, Qiqihar Medical College, Heilongjiang, China.

**Keywords:** Cell Biology, Nephrology, Hypertension, Potassium channels

## Abstract

We examine whether calcineurin or protein phosphatase 2B (PP2B) regulates the basolateral inwardly rectifying potassium channel Kir4.1/Kir5.1 in the distal convoluted tubule (DCT). Application of tacrolimus (FK506) or cyclosporine A (CsA) increased whole-cell Kir4.1/Kir5.1-mediated K^+^ currents and hyperpolarized the DCT membrane. Moreover, FK506-induced stimulation of Kir4.1/Kir5.1 was absent in kidney tubule–specific 12 kDa FK506-binding protein–knockout mice (Ks-FKBP-12–KO). In contrast, CsA stimulated Kir4.1/Kir5.1 of the DCT in Ks-FKBP-12–KO mice, suggesting that FK506-induced stimulation of Kir4.1/Kir5.1 was due to inhibiting PP2B. Single-channel patch-clamp experiments demonstrated that FK506 or CsA stimulated the basolateral Kir4.1/Kir5.1 activity of the DCT, defined by NP_o_ (a product of channel number and open probability). However, this effect was absent in the DCT treated with Src family protein tyrosine kinase (SFK) inhibitor or hydroxyl peroxide. Fluorescence imaging demonstrated that CsA treatment increased membrane staining intensity of Kir4.1 in the DCT of *Kcnj10^fl/fl^* mice. Moreover, CsA treatment had no obvious effect on phosphorylated NaCl cotransporter (pNCC) expression in Ks-Kir4.1–KO mice. Immunoblotting showed acute FK506 treatment increased pNCC expression in *Kcnj10^fl/fl^* mice, but this effect was attenuated in Ks-Kir4.1–KO mice. In vivo measurement of thiazide-induced renal Na^+^ excretion demonstrated that FK506 enhanced thiazide-induced natriuresis. This effect was absent in Ks-FKBP-12–KO mice and blunted in Ks-Kir4.1–KO mice. We conclude that inhibition of PP2B stimulates Kir4.1/Kir5.1 of the DCT and NCC and that PP2B inhibition–induced stimulation of NCC is partially achieved by stimulation of the basolateral Kir4.1/Kir5.1.

## Graphical abstract

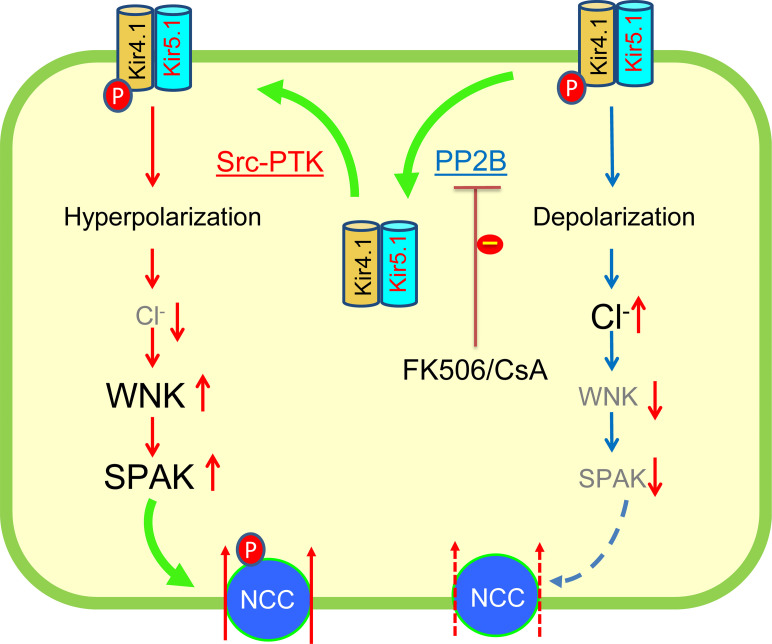

## Introduction

Expression and activity of calcineurin (protein phosphatase 2B, PP2B) are detected in the renal tubules including proximal tubule, thick ascending limb, distal convoluted tubule (DCT), and cortical collecting duct (CCD) (1–4). Acute inhibition of PP2B with cyclosporine A (CsA) or tacrolimus (FK506), 2 frequently used immunosuppressive drugs after organ transplantation (5), has been shown to stimulate cation-coupled Cl^–^ cotransporters such as NKCC2 and NCC (1, 2). Moreover, Hoorn et al. have suggested that the stimulation of NCC may be, in part, responsible for PP2B inhibition–induced hypertension and hyperkalemia (2), 2 common side effects of using CsA or FK506 (6, 7). This notion was supported by the finding that the inhibition of NCC with thiazide was able to reverse the effect of tacrolimus on hypertension (2). Several studies have suggested that PP2B may play a role in the regulation of renal K^+^ excretion and K^+^ homeostasis (2, 8, 9). Our previous study demonstrated that decreased dietary K^+^ intake suppressed PP2B catalytic subunits’ expression in the rat kidney (8). Moreover, Uchida et al. have shown that the acute inhibition of PP2B with tacrolimus abolished acute high K^+^ intake–induced (HK-induced) inhibition of NCC (9). It is now well established that the NCC activity not only is responsible for the reabsorption of 5% filtered Na^+^ load but also plays a critical role in the regulation of epithelial Na^+^ channel–dependent (ENaC-dependent) renal K^+^ excretion by controlling Na^+^ delivery to the aldosterone-sensitive distal nephron (ASDN) (10, 11), because Na^+^ delivery rate to the ASDN is an important factor determining ENaC-dependent renal K^+^ excretion (12, 13). Therefore, PP2B may play a physiological role in regulating renal K^+^ excretion by controlling NCC expression/activity. A large body of evidence has demonstrated that the basolateral inwardly rectifying potassium channel Kir4.1/Kir5.1 of the DCT determines the expression and activity of NCC (14–17). For instance, high Kir4.1/Kir5.1 activity is associated with increased NCC expression/activity during decreased dietary K^+^ or Na^+^ intakes whereas low Kir4.1/Kir5.1 activity is associated with decreased NCC expression/activity during increased dietary K^+^ or Na^+^ intakes (14–17). Thus, the aim of the present study is to test whether PP2B activity may also regulate the basolateral Kir4.1/Kir5.1 activity in the DCT and whether acute calcineurin inhibition–induced stimulation of NCC activity is, in part, achieved by the augmentation of the basolateral Kir4.1/Kir5.1 activity.

## Results

**Figure 1:**
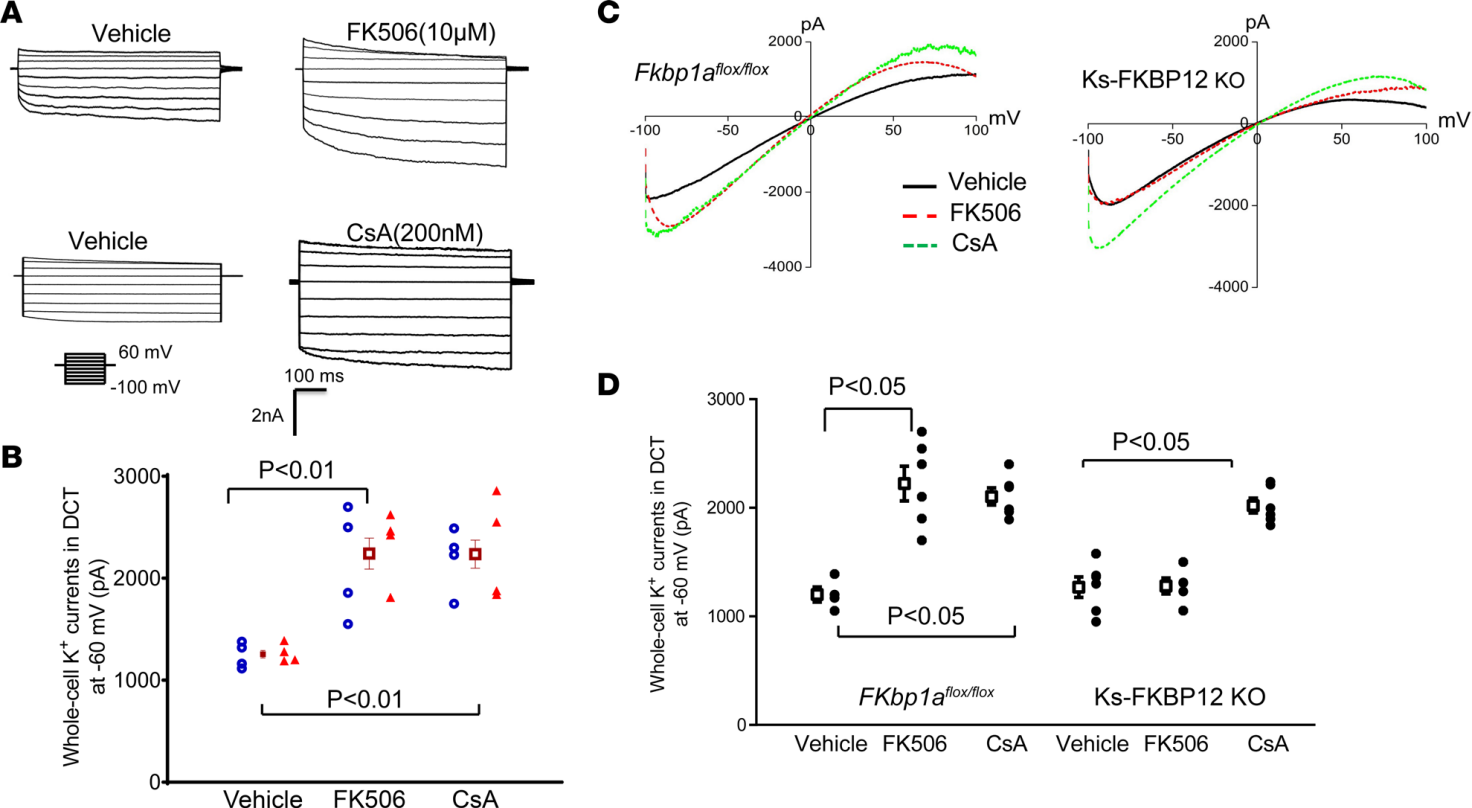
Inhibition of calcineurin (PP2B) increases the basolateral K^+^ currents in the DCT. (**A**) A set of whole-cell recordings shows the effect of FK506 (10 μM) and CsA (200 nM) on Kir4.1/Kir5.1-mediated K^+^ currents in the DCT. The Kir4.1/Kir5.1-mediated whole-cell K^+^ currents were measured with a step protocol from −100 to 60 mV. The vehicle, FK506, or CsA was added in the bath for 10 minutes. (**B**) A scatterplot summarizing the results of above experiments in which Ba^2+^-sensitive K^+^ currents (Kir4.1/Kir5.1) of the DCT were measured at −60 mV. Each data point from male (blue circles) or female mice (red triangles) is presented for 2 separate columns, and the mean value ± SEM (including data from male and female mice) is shown in the middle. Significance is determined by 1-way ANOVA. (**C**) A set of recordings shows Kir4.1/Kir5.1-mediated whole-cell K^+^ currents in DCT of male *Fkbp1a^fl/fl^* and male Ks-FKBP12–KO mice treated with FK506 (0.75 mg/kg BW) or CsA (3 mg/kg BW) by peritoneal injection 30 minutes before the experiment, respectively. The whole-cell K^+^ currents were measured with a ramp protocol from −100 to 100 mV. (**D**) A scatterplot summarizing the results of experiments in which Kir4.1/Kir5.1-mediated whole-cell K^+^ currents of the DCT were measured at −60 mV. Mean values and SEM are shown on the left of each column. A symmetric 140 mM KCl solution was used for the bath and the pipette. Significant difference as determined by 2-way ANOVA.

We first examined the effect of tacrolimus (FK506) and cyclosporine A (CsA) on the basolateral K^+^ channel activity of the DCT using whole-cell patch-clamp recording technique by measuring Ba^2+^-sensitive whole-cell K^+^ currents in the early part of the DCT (DCT1). The reason to conduct experiments in the DCT1 is due to the fact that no K^+^ channel other than Kir4.1/Kir5.1 heterotetramer is detected in DCT1 and Kir4.1/Kir5.1 heterotetramer is the predominant form of K^+^ channel in the basolateral membrane (18–20). Thus, the whole-cell K^+^ currents of DCT1 represent Kir4.1/Kir5.1 activity. Figure 1A is a set of whole-cell recordings showing the Ba^2+^-sensitive K^+^ currents measured with step protocol from –100 to 60 mV in the DCT1 treated with vehicle, FK506 (10 μM), or CsA (200 nM) for 10 minutes. Figure 1B is a scatterplot summarizing the results in 3 male (m) and 3 female (f) mice (measured at –60 mV) demonstrating that acute addition of FK506 or CsA increased the whole-cell K^+^ currents from 1,283 ± 62 pA to 2,340 ± 135 pA for FK506 and 2,240 ± 140 pA for CsA. We next examined the effect of FK506 on the basolateral K^+^ channels in the DCT1 of kidney tubule–specific 12 kDa FK506-binding protein–knockout mice (Ks-FKBP-12–KO) and *Fkbp1a^fl/fl^* mice (control) treated in vivo with FK506 or CsA. The reasoning is to examine whether in vivo treatment of CsA or FK506 could also stimulate Kir4.1/Kir5.1 of the DCT and to examine whether FK506-induced stimulation of Kir4.1/Kir5.1 was due to the inhibition of PP2B. The male control and male Ks-FKBP12–KO mice were treated with FK506 (0.75 mg/kg BW) by peritoneal injection (p.i.) 30 minutes before experiments. Figure 1C is a recording showing the Ba^2+^-sensitive whole-cell K^+^ currents in the DCT1 of the control mice and Ks-FKBP12–KO mice treated with vehicle or FK506, and the results are summarized in Figure 1D. It is apparent that Kir4.1/Kir5.1-mediated K^+^ currents of the DCT1 were significantly larger (2,220 ± 160 pA, *n* = 6) in FK506-treated group than vehicle-treated *Fkbp1a^fl/fl^* mice (1,200 ± 70 pA, *n* = 4). However, K^+^ currents in FK506-treated Ks-FKBP12–KO mice (1,280 ± 70 pA, *n* = 5) were similar to the vehicle-treated group (1,270 ± 100 pA, *n* = 6), suggesting that FK506-induced inhibition of PP2B is achieved by binding to FKBP12. In contrast, CsA treatment (3 mg/kg BW by p.i. 30 minutes before experiments) still significantly increased the whole-cell K^+^ currents in both control (2,100 ± 80 pA, *n* = 6) and Ks-FKBP12–KO mice (2,020 ± 80 pA, *n* = 6) (Figure 1, C and D). This suggests that the CsA-induced stimulation of Kir4.1/Kir5.1 of the DCT was not affected by deleting FKBP12 because CsA inhibits PP2B by binding to cyclophilin (21).

**Figure 2:**
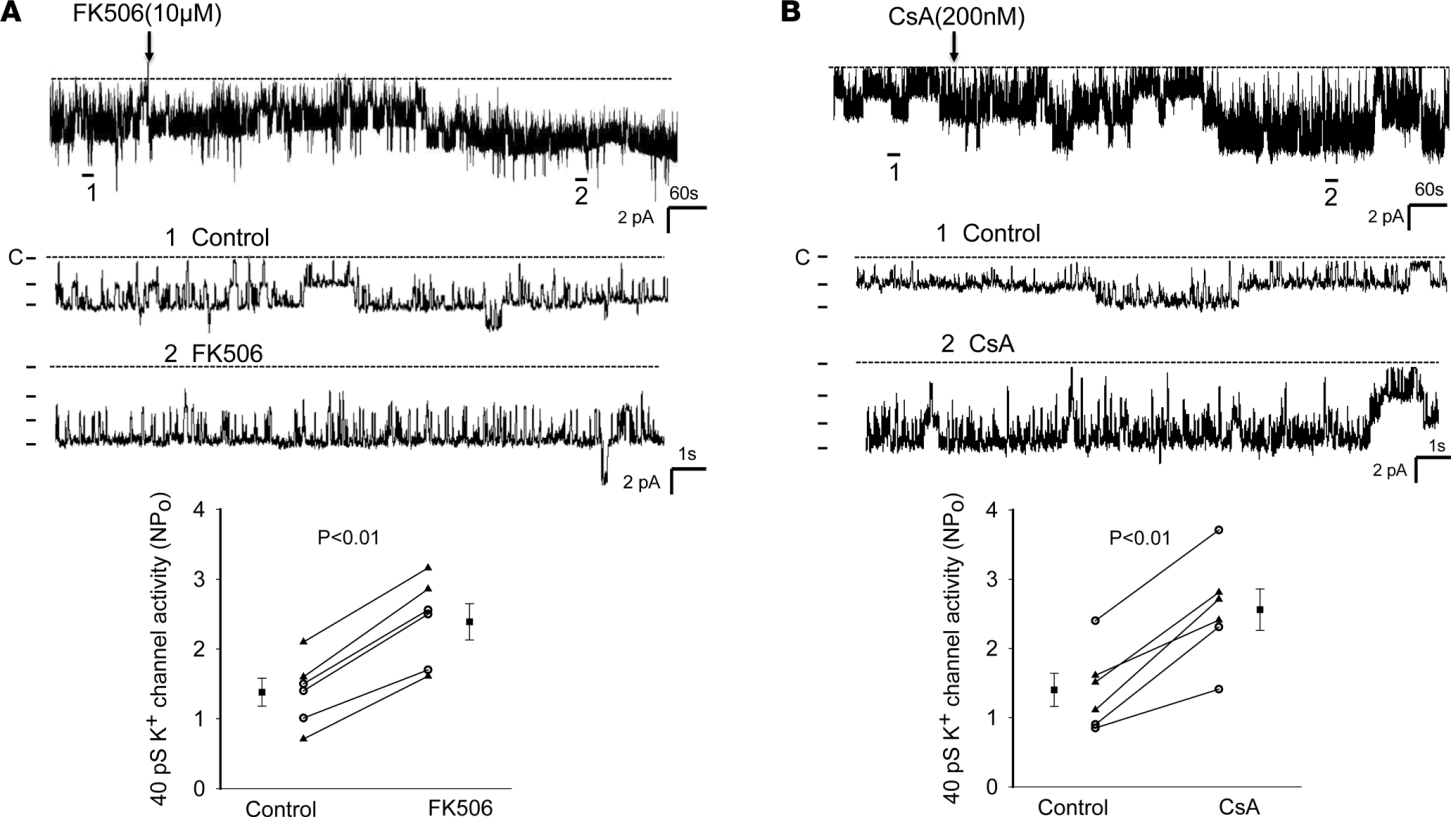
Inhibition of calcineurin (PP2B) stimulates the basolateral Kir4.1/5.1 (40 pS K^+^) channels in the DCT. A single-channel recording shows the effect of 10 μM FK506 (**A**) or 200 nM CsA (**B**) on the basolateral 40 pS K^+^ channels in DCT. Two parts of the trace (indicated by numbers) are extended to show the fast resolution. The experiments were performed in cell-attached patches. The results of the experiments are summarized in 2 scatterplots at the bottom of each panel. The DCT was bathed in a solution containing 140 mM NaCl/5 mM KCl, and the pipette solution contained 145 mM K^+^. Significance is determined by a paired Student’s *t* test. Data from male and female mice are indicated by circles and triangles, respectively. Mean value (including male and female mice) is indicated by a square.

**Figure 3:**
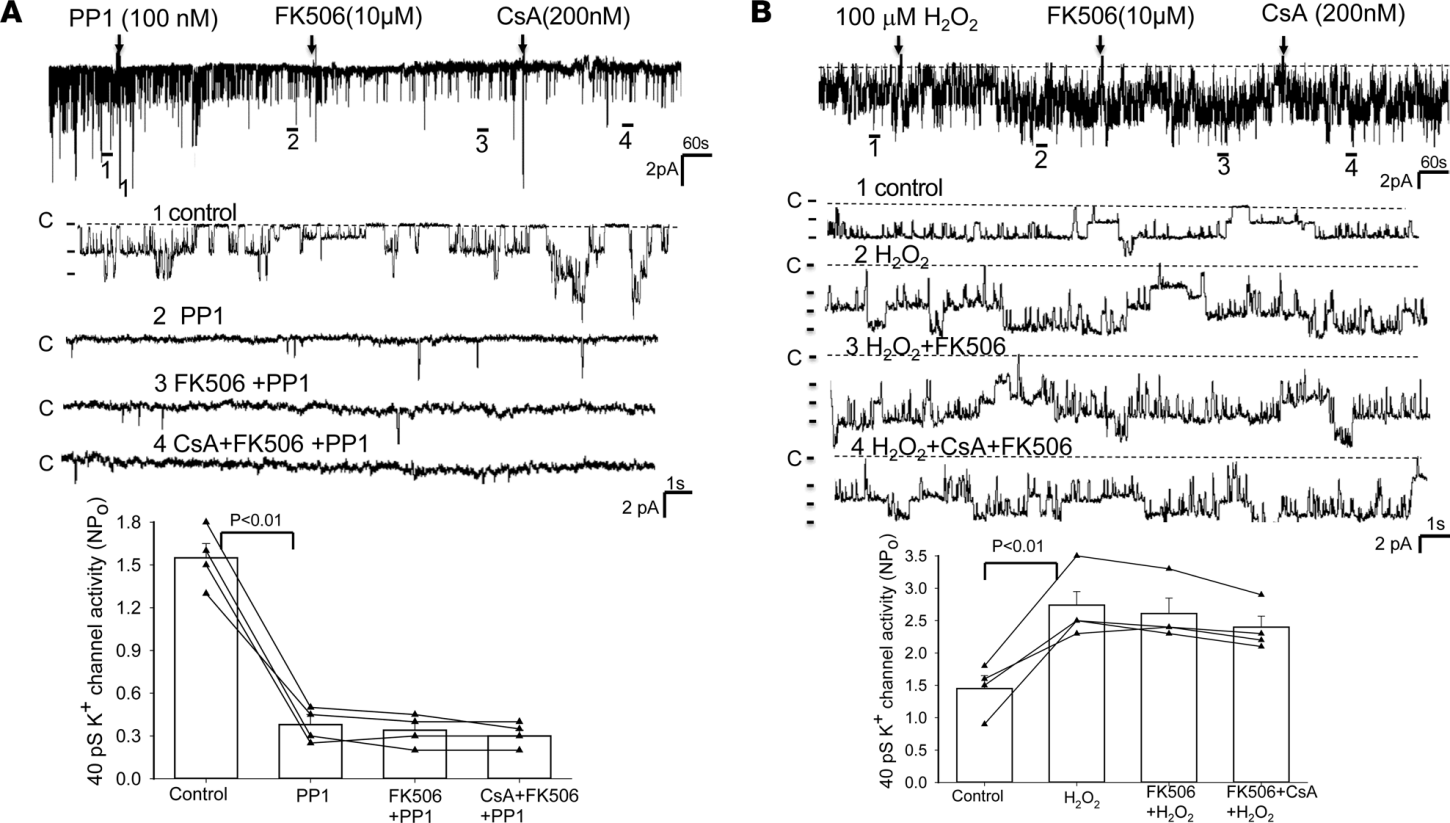
The effect of calcineurin inhibitors on Kir4.1/Kir5.1 is absent in the DCT treated with PP1 or H_2_O_2_. A single-channel recording demonstrates the effect of FK506 and CsA on the 40 pS K^+^ channels in the DCT pretreated with 100 nM PP1, an inhibitor of SFK (**A**) or pretreated with 100 μm H_2_O_2_ (**B**). Four parts of the trace (indicated by numbers) are extended to show the fast resolution. The experiments were performed in cell-attached patches (male mice). The results of the experiments are summarized in 2 bar graphs with scatterplots at the bottom of each panel. The DCT was bathed in a solution containing 140 mM NaCl/5 mM KCl, and the pipette solution contained 145 mM K^+^. Significance is determined by 1-way ANOVA.

The notion that the inhibition of PP2B stimulates Kir4.1/Kir5.1 of the DCT is also suggested by testing the effects of FK506 and CsA on the 40 pS K^+^ channels (a Kir4.1/Kir5.1 heterotetramer) of the isolated DCT using single channel recording. Figure 2A shows a single-channel recording showing the effect of FK506 (10 μM) on the basolateral 40 pS K^+^ channel (Kir4.1/Kir5.1). Addition of FK506 stimulated the 40 pS K^+^ channel activity in the DCT and increased the NP_o_ (a product of channel number and open probability) from 1.38 ± 0.20 to 2.39 ± 0.26 (3m/3f mice). We have also tested the effect of CsA on the 40 pS K^+^ channels of the DCT, and Figure 2B is a single-channel recording showing that adding CsA (200 nM) also stimulated Kir4.1/Kir5.1 of the DCT and increased channel activity from 1.40 ± 0.24 to 2.56 ± 0.25 (3m/3f mice). Previous study demonstrated that Kir4.1/Kir5.1 activity was modulated by Src family protein tyrosine kinase (SFK), which phosphorylated Kir4.1 protein, thereby increasing Kir4.1/Kir5.1 activity in the DCT (19). To test whether the stimulatory effect of FK506 or CsA on Kir4.1/Kir5.1 was due to enhancing SFK-induced stimulation of Kir4.1/Kir5.1 by inhibiting dephosphorylation of the K^+^ channel, we examined the effect of FK506 or CsA on Kir4.1/Kir5.1 in the DCT treated with PP1 (22), a specific SFK inhibitor. Figure 3A is a single-channel recording demonstrating that the inhibition of SFK with PP1 not only decreased the 40 pS K^+^ channel NP_o_ (from 1.55 ± 0.10 to 0.38 ± 0.06, *n* = 4) but also abolished the effect of FK506 (NP_o_, 0.34 ± 0.06, *n* = 4) or CsA (NP_o_, 0.30 ± 0.06, *n* = 4) on the Kir4.1/Kir5.1 activity in the DCT. This suggests the possibility that PP2B may be involved in regulating the Kir4.1/Kir5.1 activity of the DCT by dephosphorylating Kir4.1/Kir5.1 induced by SFK. This notion is further supported by experiments in which we have examined whether CsA or FK506 is able to stimulate Kir4.1/Kir5.1 in the DCT treated with H_2_O_2_, which is known to stimulate SFK (23). Figure 3B is a single-channel recording made in a cell-attached patch showing that adding 100 μM H_2_O_2_ increased the Kir4.1/Kir5.1 channel activity (NP_o_) from 1.45 ± 0.2 to 2.7 ± 0.27 (*n* = 4 male mice). Moreover, adding 10 μM FK506 or CsA (200 nM) had no additional effect on the 40 pS K^+^ channel (FK506, 2.6 ± 0.24 and CsA, 2.4 ± 0.17, *n* = 4). This supports the hypothesis that PP2B inhibitor–induced activation of Kir4.1/Kir5.1 of the DCT is related to directly or indirectly modulated SFK-induced phosphorylation of the K^+^ channel of the DCT.

**Figure 4:**
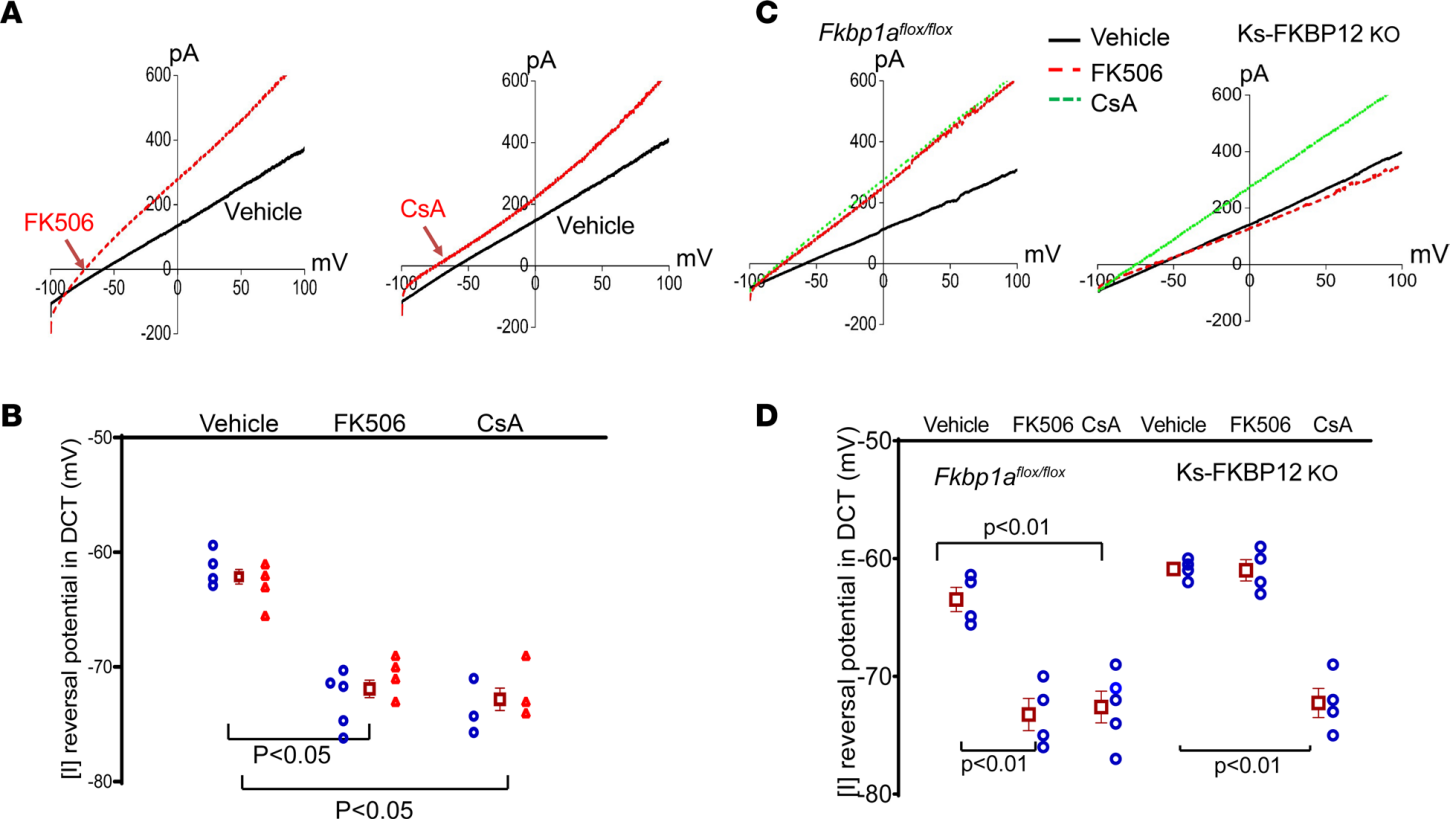
Inhibition of calcineurin (PP2B) hyperpolarizes the DCT membrane. (**A**) A set of traces of the whole-cell voltage-clamp shows the effect of FK506 and CsA on [I] reversal potential in the DCT of the mice treated with vehicle, FK506 (0.75 mg/kg BW) or CsA (3 mg/kg BW) by p.i. 30 minutes before experiments. (**B**) A scatterplot summarizing the results of the experiments in which [I] reversal potential of the DCT was measured with whole-cell recording. Each data point from male (blue circles) or female mice (red triangles) is presented for 2 separate columns, and the mean value ± SEM (including data from male and female mice) is shown in the middle. Significance is determined by 1-way ANOVA. (**C**) A set of traces of the whole-cell voltage-clamp shows the effect of FK506 and CsA on [I] reversal potential in the DCT of male *Fkbp1a^fl/fl^* mice or Ks-FKBP12–KO mice treated with vehicle, FK506 (3 mg/kg BW) or CsA (3 mg/kg BW) by p.i. 30 minutes before experiments. (**D**) A scatterplot summarizing the results of above experiments (male mice), and mean values and SEM are shown on the left of each column. Significance is determined by 2-way ANOVA. The DCT was bathed in a solution containing 140 mM NaCl/5 mM KCl, and the pipette solution contained 145 mM K^+^.

Because Kir4.1/Kir5.1 is the predominant form of K^+^ channels in the basolateral membrane of the DCT (20), we expect that the stimulation of Kir4.1/Kir5.1 by calcineurin inhibitors should increase the negativity of DCT membrane potential (hyperpolarization). Thus, we next examined the effect of FK506 and CsA on the reversal potential of inward-to-outward current ([I] reversal potential), which is an index of the membrane potential. (In our previous study we also refer to it as I_K_ reversal potential.) Figure 4A shows 2 whole-cell recordings demonstrating the [I] reversal potential of the DCT treated with vehicle, FK506 (0.75 mg/kg BW), or CsA (3 mg/kg BW) by p.i. 30 minutes before experiments. Figure 4B is a scatterplot summarizing the results of experiments performed in both male and female mice. FK506 treatment increased negativity of [I] reversal potential of the DCT from –62 ± 1 mV to –72.5 ± 1 mV (3m/3f mice). CsA treatment increased negativity of [I] reversal potential of the DCT from –62 ± 1 mV to –73 ± 1 mV (3m/3f mice). Thus, the inhibition of PP2B hyperpolarized the membrane potential of the DCT. We have also examined the effect of high-dose FK506 (3 mg/kg BW by p.i. 30 minutes before experiments) on [I] reversal potential in *Fkbp1a^fl/fl^* mice and Ks-FKBP12–KO mice. From the inspection of Figure 4, C and D, it is apparent that high-dose FK506 hyperpolarized the DCT membrane (control value, –63 ± 1 mV; FK506, –73 ± 1 mV, *n* = 4 male mice) to the same extent as the low dose. Moreover, this effect was abolished in Ks-FKBP12 KO mice (control value, –61 ± 1 mV; FK506, –61 ± 1 mV, *n* = 4 male mice), suggesting FK506-induced hyperpolarization was due to the inhibition of PP2B because it required FKBP12. In contrast, CsA hyperpolarized the DCT membrane not only in control mice (–72.5 ± 1 mV, *n* = 5 male mice) but also in Ks-FKBP12–KO mice (–72 ± 1 mV, *n* = 4 male mice).

**Figure 5:**
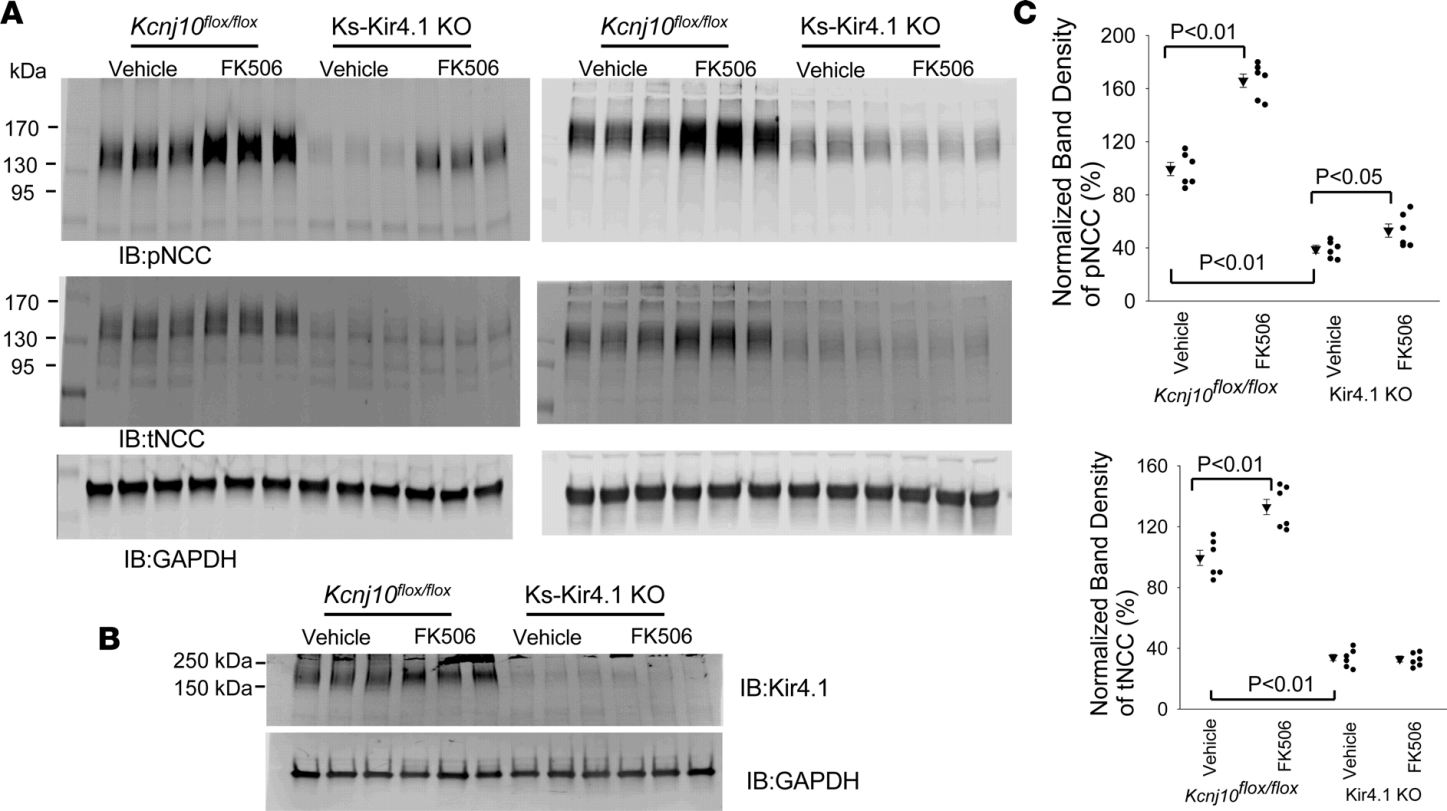
FK506 treatment–induced stimulation of pNCC expression is attenuated in Ks-Kir4.1–KO mice. (**A**) Two Western blots show the abundance of pNCC^T53^ and tNCC in male *Kcnj10^fl/fl^* mice and in Ks-Kir4.1–KO mice treated with vehicle (control) and FK506 (0.75 mg/kg BW). FK506 or vehicle was applied by peritoneal injection 30 minutes before the experiment. (**B**) A Western blot shows the expression of Kir4.1 in the control and Ks-Kir4.1–KO mice to validate the deletion of Kir4.1. Kir4.1 band represents a Kir4.1 homotetramer and Kir4.1/Kir5.1 heterotetramer. (**C**) The normalized band density of pNCC and tNCC expression from the experiments is summarized in 2 scatterplots. For the Western blot experiments, the lysate obtained from the renal cortex tissue was unheated. Significance was determined by 2-way ANOVA.

The inhibition of PP2B has been demonstrated to increase the abundance of phosphorylated NaCl cotransporter (pNCC) and total NaCl cotransporter (tNCC) (2). Since a high basolateral K^+^ channel activity is associated with increased NCC function (15, 24), we speculated that PP2B inhibition–induced stimulation of Kir4.1/Kir5.1 in the DCT may contribute partially to PP2B inhibition–induced stimulation of NCC. Thus, we next examined the effect of FK506 treatment (0.75 mg/kg BW by p.i. 30 minutes before experiments) on the expression of pNCC and tNCC in the control (*Kcnj10^fl/fl^*) and in Ks-Kir4.1–KO mice. Figure 5A shows 2 Western blots from 6 male mice demonstrating the expression of pNCC and tNCC in the control (*Kcnj10^fl/fl^*) and Ks-Kir4.1–KO mice treated with FK506 or vehicle (uncut Western blot is shown in Supplemental Figure 1; supplemental material available online with this article; https://doi.org/10.1172/jci.insight.165987DS1). Figure 5B is a Western blot showing the Kir4.1 expression to validate the deletion of Kir4.1. Figure 5C shows 2 scatterplots summarizing the normalized band density of pNCC and tNCC. We verified the previous finding that FK506 treatment robustly increases the expression of pNCC (by 66% ± 5%) (2). Moreover, FK506 treatment also slightly increased tNCC (by 33% ± 6%), an effect possibly induced by inhibiting NCC ubiquitination after increasing NCC phosphorylation (25). We confirmed our previous observations that pNCC and tNCC expression in Kir4.1-KO mice decreased to 39% ± 3% and 34% ± 2% of the control value observed in *Kcnj10^fl/fl^* mice, respectively (14, 16). Moreover, we observed that the effect of FK506 treatment on pNCC expression was attenuated or even largely abolished in Kir4.1-deficient mice. By calculation of these results, we observed that mean pNCC expression of Kir4.1-KO mice treated with FK506 was only 53% ± 5% of the control value. Thus, FK506 treatment increased pNCC expression in Kir4.1-KO mice only by 35% compared with vehicle whereas it was 66% in *Kcnj10^fl/fl^* mice. Thus, calcineurin inhibitor–induced stimulation of pNCC was substantially attenuated in Kir4.1-KO mice. This suggests that the effect of PP2B inhibition–induced stimulation of pNCC was at least in part through activation of the basolateral Kir4.1/Kir5.1 of the DCT.

**Figure 6:**
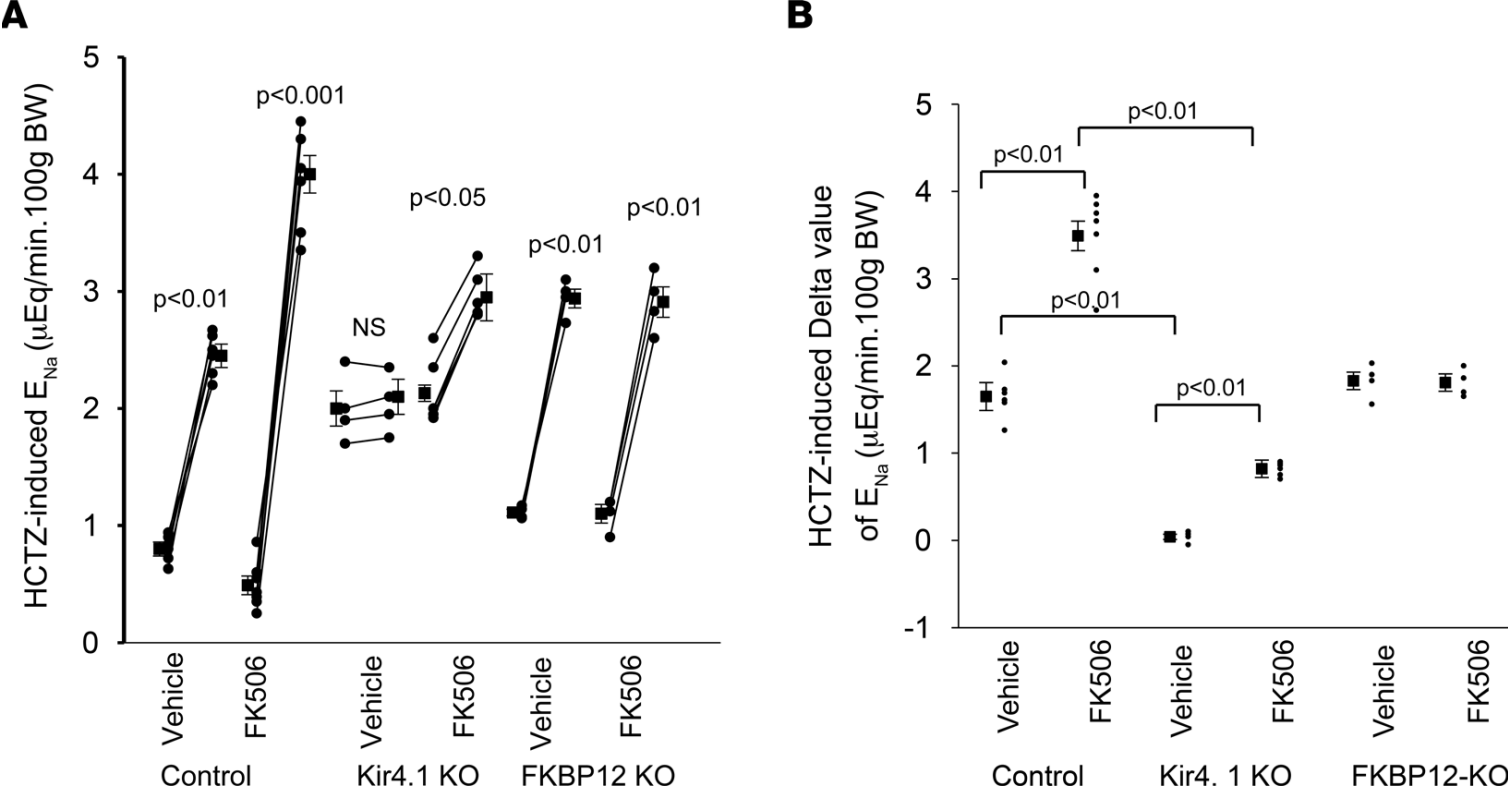
FK506-induced stimulation of HCTZ-induced natriuresis is attenuated in Ks-Kir4.1–KO mice and is absent in Ks-FKBP12–KO mice. (**A**) A line graph shows the results of each experiment in which urinary sodium excretion (E_Na_) was measured before and after a single dose of hydrochlorothiazide (HCTZ; 30 mg/kg BW) in control mice (*Kcnj10^fl/fl^* and *Fkbp1a^fl/fl^*), Ks-Kir4.1–KO mice, and Ks-FKBP12–KO mice with or without FK506 (0.75 mg/kg BW) injection for 30 minutes before the experiment. The significance is determined by paired *t* test. (**B**) A scatterplot shows the mean value and each single data point of the net HCTZ-induced E_Na_ in different groups. Significance was determined by 2-way ANOVA.

The finding that acute FK506 treatment increased NCC activity in the control mice but to a lesser degree in Ks-Kir4.1-KO mice was also confirmed by in vivo measurement of hydrochlorothiazide-induced **(**HCTZ-induced) (30 mg/kg BW) urinary Na^+^ excretion (E_Na_) after initial saline perfusion. Figure 6A summarizes results of each individual experiment in which HCTZ-induced E_Na_ was measured in the control mice (3 male *Kcnj10^fl/fl^* and 3 male *Fkbp1a^fl/fl^*), Ks-Kir4.1–KO mice (*n* = 5 male), and Ks-FKBP12–KO mice (*n* = 4 male) treated with FK506 (0.75 mg/kg BW by p.i.) or vehicle 30 minutes before experiments. Figure 6B is a scatterplot summarizing the delta value of HCTZ-induced E_Na_ in the control, Ks-Kir4.1–KO, and Ks-FKBP12–KO mice treated with vehicle or FK506. HCTZ-induced natriuresis in FK506-treated control mice (0.49 ± 0.08 to 4.01 ± 0.16 μEq/min/100 g BW, *n* = 6 male mice) was significantly larger than in vehicle-treated control (0.80 ± 0.06 to 2.45 ± 0.10 μEq/min/100 g BW, *n* = 6 male mice). Deletion of Kir4.1 inhibited NCC as evidenced by the fact that baseline E_Na_, which was measured after initial saline infusion (0.3 mL), was significantly higher than the control mice (14). Moreover, HCTZ-induced E_Na_ was largely absent (2.0 ± 0.15 to 2.1 ± 0.15 μEq/min/100 g BW) in 4 male Ks-Kir4.1–KO mice treated with vehicle. However, FK506 treatment was still able to slightly increase HCTZ-induced E_Na_ (2.13 ± 0.07 to 2.95 ± 0.20 μEq/min/100 g BW, *n* = 5 male mice). But, the delta value (0.82 ± 0.10 μEq/min/100 g BW) in the FK506-treated Ks-Kir4.1–KO mice was significantly smaller than those in FK506-treated control mice (3.49 ± 0.17 μEq/min/100 g BW). Again, the stimulatory effect of FK506 on HCTZ-induced E_Na_ (FK506, 1.10 ± 0.08 to 2.91 ± 0.13 μEq/min/100 g BW, *n* = 4 male mice) was completely absent in Ks-FKBP12–KO mice (vehicle, 1.11 ± 0.03 to 2.94 ± 0.08 μEq/min/100 g BW, *n* = 4 male mice). Thus, data suggest that FK506-induced stimulation of E_Na_ requires FKBP12 and is partially achieved by activation of Kir4.1/Kir5.1 of the DCT. Acute FK506 treatment did not affect the baseline renal K^+^ excretion (E_K_) in comparison to vehicle (Supplemental Figure 1A). However, FK506 treatment increased HCTZ-dependent renal E_K_, from 0.57 ± 0.03 μEq/min/100 g BW (vehicle) to 0.8 ± 0.06 μEq/min/100 g BW (*n* = 5 male mice) (Supplemental Figure 2B). This is presumably induced by increasing Na^+^ delivery to the late distal tubule thereby enhancing ENaC-dependent E_K_. However, HCTZ-induced E_Na_/E_K_ ratio in FK506-treated mice was higher than in the vehicle-treated group (Supplemental Figure 3). This suggests that acute inhibition of calcineurin mainly stimulates NCC of the DCT but it had lesser effect on ENaC.

**Figure 7:**
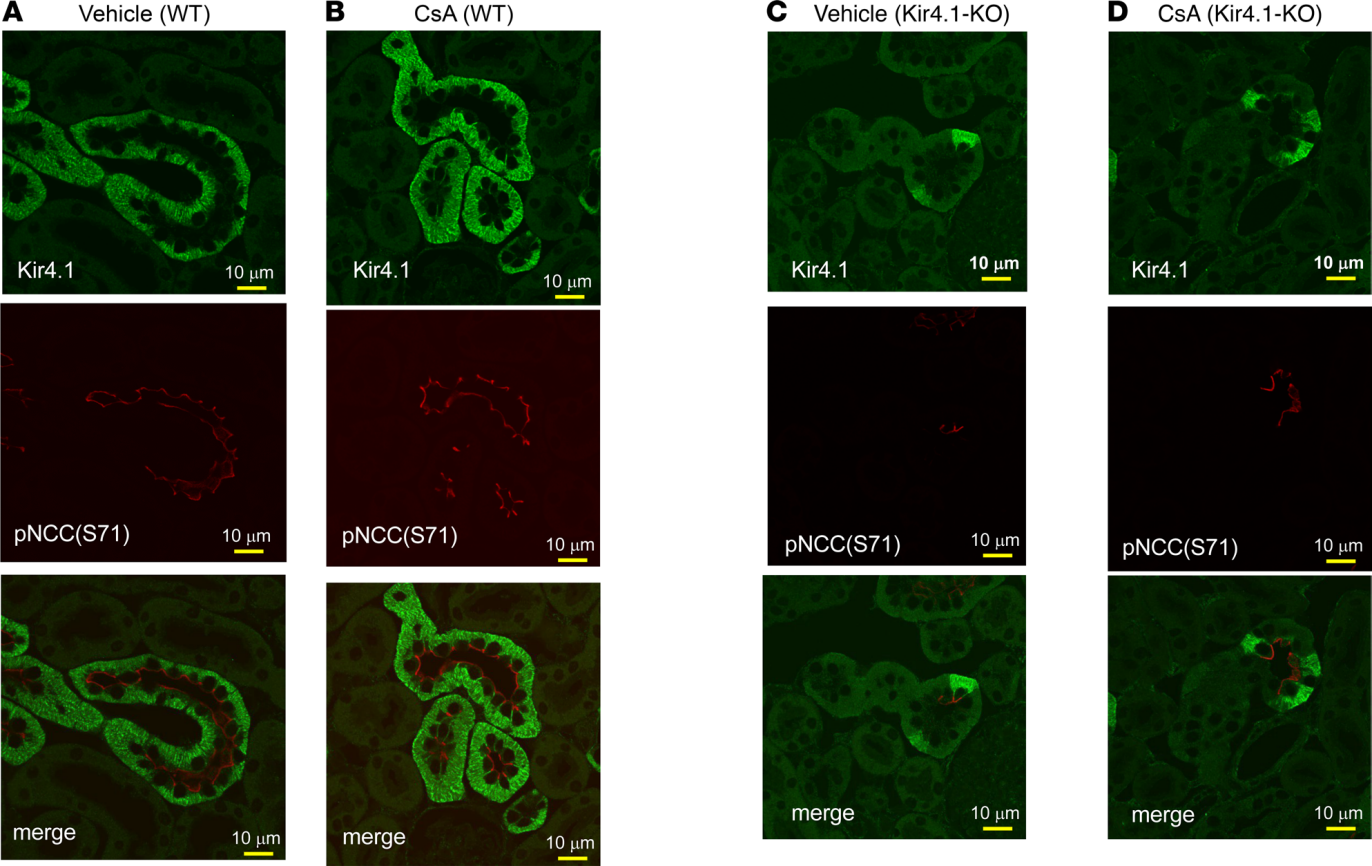
CsA treatment increases immunostaining intensity of Kir4.1. Immunostaining image shows the expression of Kir4.1 and pNCC (S71) in the DCT of *Kcnj10^fl/fl^* mice treated with the vehicle (**A**) or CsA (**B**) and Ks-Kir4.1–KO mice treated with the vehicle (**C**) or CsA (**D**). The mice were treated with the vehicle or CsA (3 mg/kg BW) by peritoneal injection 30 minutes before the perfusion fixation of the kidneys.

We next used immunofluorescence microscopy to examine the basolateral surface expression of Kir4.1 and the luminal surface expression of pNCC (Ser71) in *Kcnj10^fl/fl^* (control) treated with vehicle (Figure 7A) or CsA (3 mg/kg BW by p.i. 30 minutes before perfusion fixation) (Figure 7B) and in Ks-Kir4.1–KO mice treated with vehicle (Figure 7C) or CsA (Figure 7D). Semiquantification of the Kir4.1 image showed that CsA treatment increased basolateral Kir4.1 fluorescence intensity compared with vehicle-treated animals by 45% (*P* < 0.05) from 76 ± 10 to 110 ± 11 (arbitrary units) (Supplemental Figure 3C). Although apical pNCC immunostaining in Kir4.1-KO mice was faint, a clear luminal staining of pNCC was still obvious in the DCT cells showing Kir4.1 expression remained (because of incomplete deletion). In contrast, Kir4.1-deficient DCT cells showed virtually no pNCC fluorescence staining. The results are consistent with the Western blot and in vivo measurement of HCTZ-induced natriuresis. It is possible that the increase in pNCC may occur only in the DCT cells in which Kir4.1 is still present. This suggests that Kir4.1 activity is required for calcineurin inhibition–induced stimulation of NCC.

## Discussion

The main finding of the present study is that acute application of calcineurin inhibitors increased the basolateral Kir4.1/Kir5.1 activity of the DCT. Two lines of evidence have strongly suggested that the FK506 or CsA-induced stimulation of Kir4.1/Kir5.1 activity is due to the inhibition of PP2B: 1) CsA and FK506, 2 calcineurin inhibitors with different structures, had the same stimulatory effect on the Kir4.1/Kir5.1; and 2) the effect of FK506 on the basolateral Kir4.1/Kir5.1 was completely absent in the FKBP12-deficient mice while CsA was still able to stimulate the K^+^ channel, suggesting that the effect of FK506 but not CsA was achieved by binding to FKBP12 thereby inhibiting PP2B (21, 26). The notion that PP2B regulates the basolateral Kir4.1/Kir5.1 of the DCT is supported by 4 lines of evidence: 1) application of FK506 and CsA increased the basolateral 40 pS K^+^ channel activity defined by NP_o_; 2) the Kir4.1/Kir5.1-mediated whole-cell K^+^ currents were larger in the DCT treated with FK506 and CsA than with vehicle-treated tubule; 3) CsA treatment increased the membrane staining intensity of Kir4.1; and 4) FK506 and CsA treatment increased the negativity of the [I] reversal potential of the DCT, an indication of hyperpolarization. The role of PP2B in the regulation of membrane transport of the DCT has been well established by the finding that the acute inhibition of PP2B increased NCC expression/activity (1, 2, 9). Thus, our present results have suggested that PP2B regulates the membrane transport not only by modulating NCC but also by regulating the basolateral K^+^ channels of the DCT. Although the present study has demonstrated that calcineurin inhibitors stimulate the basolateral Kir4.1/Kir5.1 activity in the DCT, it is not known whether CsA or FK506 is also able to regulate Kir4.1 homotetramer because Kir4.1 homotetramer, a 20–25 pS K^+^ channel, is hard to detect under control conditions (18, 27, 28). Thus, a separate study may be required using Kir5.1-KO mice to determine the effect of CsA or FK506 on Kir4.1 homotetramer.

Although the mechanism by which PP2B regulates Kir4.1/Kir5.1 is not completely understood, 2 lines of evidence suggest the possibility that PP2B regulates Kir4.1/Kir5.1 by targeting SFK-mediated tyrosine phosphorylation. First, the inhibition of SFK abolished the effect of FK506 or CsA on the basolateral Kir4.1/Kir5.1 activity. Second, CsA or FK506 had no additional effect on Kir4.1/Kir5.1 of the DCT treated with H_2_O_2_. Our previous study has demonstrated that Kir4.1 protein is a substrate of SFK, which phosphorylates Kir4.1 at its N-terminus (19). The role of SFK in regulating Kir4.1/Kir5.1 was also supported by in vitro and in vivo studies in which we have demonstrated that SFK increased Kir4.1/Kir5.1-mediated K^+^ currents in the DCT and the surface expression (19, 29). Thus, we speculate that CsA or FK506-induced activation of Kir4.1/Kir5.1 may be achieved by inhibiting PP2B-dependent dephosphorylation of Kir4.1/Kir5.1 thereby enhancing the effect of SFK on the basolateral K^+^ channels of the DCT. Although calcineurin is considered a protein serine/threonine phosphatase, a previous study has demonstrated that PP2B was also able to dephosphorylate tyrosine phosphorylation; however, *K_m_* and *V_max_* values were lower for phosphotyrosyl substrates than phosphoseryl substrates (30). Moreover, PP2B has been reported to regulate tyrosine phosphorylation of Cl^–^ channels in renal epithelial cells (31). Further experiments are required to explore whether PP2B is able to modulate tyrosine phosphorylation of Kir4.1/Kir5.1.

We have also verified the previous finding that the acute inhibition of PP2B increased NCC expression/activity (2, 32). However, 3 lines of evidence strongly suggest that the PP2B inhibition–induced stimulation of NCC is at least in part through stimulation of Kir4.1/Kir5.1: 1) FK506-induced stimulation of both pNCC and tNCC expression was attenuated in Ks-Kir4.1–KO mice; 2) HCTZ-induced natriuresis was also smaller in Ks-Kir4.1–KO mice than *Kcnj10^fl/fl^* mice; 3) CsA-induced stimulation of apical pNCC expression was more obvious in the DCT cells where Kir4.1 expression was still visible than DCT cells where Kir4.1 staining was absent. We speculate that PP2B may regulate NCC expression/activity by Kir4.1/Kir5.1-dependent and independent mechanisms. It is now well established that the basolateral Kir4.1/Kir5.1 determines the expression and activity of NCC by modulating the basolateral membrane potential (33, 34). Because the basolateral membrane potential provides the driving force for Cl^–^ ions’ movement across the basolateral membrane (35), a hyperpolarization should stimulate Cl^–^ exit thereby decreasing the intracellular Cl^–^ [Cl^–^_i_] concentrations whereas a depolarization is expected to have an opposite effect on [Cl^–^_i_] . Decreased [Cl^–^_i_] concentrations should stimulate Cl^–^-sensitive with-no-lysine kinase (WNK) such as WNK1 and WNK4 (36). WNKs are upstream protein kinases that stimulate Ste20-related protein proline/alanine-rich kinase (SPAK) and oxidative responsive kinase (OSR) (37–41). Because SPAK and OSR are responsible for stimulating NCC activity by promoting serine/threonine phosphorylation, the stimulation of WNKs induced by the activation of Kir4.1/Kir5.1 is expected to increase NCC activity/expression. PP2B may regulate the basolateral Kir4.1/Kir5.1 activity by inhibiting SFK-mediated tyrosine phosphorylation thereby decreasing the negativity of the DCT membrane potential (depolarization). Thus, the acute inhibition of PP2B with FK506 or CsA should hyperpolarize DCT membrane potential thereby decreasing [Cl^–^_i_] concentrations. The finding that acute effect of FK506 on NCC expression/activity is attenuated in Kir4.1-KO mice supports this argument. However, the finding that inhibition of PP2B was still able to increase NCC expression/activity in Kir4.1-KO mice indicates that PP2B may also regulate NCC phosphorylation directly by a Kir4.1/Kir5.1-independent mechanism.

Although the role of PP2B in the regulation of NCC and renal E_K_ has been strongly suggested by previous studies, these results were obtained using pharmacological approaches (1, 2, 9). A recent elegant study has employed a mouse model with specific deletion of calcineurin regulatory subunit B1 (CnB1) in the DCT to examine the role of PP2B in regulating NCC and renal E_K_ (42). While confirming that the inhibition of PP2B with FK506 stimulated pNCC expression in the control mice, Banki et al. have demonstrated that the expression of pNCC and tNCC was actually decreased in the mice with DCT-specific disruption of CnB1 in comparison with the corresponding control mice (42). Moreover, although the urinary K^+^ and Na^+^ excretions were compromised in DCT-specific CnB1-KO mice treated with acute (60 minutes) K^+^ loading in comparison with the corresponding control mice, the different response to K^+^ loading between 2 genotypes was absent during a prolonged K^+^ loading. This suggests that PP2B signaling in the DCT may not be indispensable for stimulating renal E_K_ during long-term HK intake. The discrepancy between Banki’s study and previous studies may be a time-dependent effect because Banki’s study was performed in the mice 3 or 10 weeks after the disruption of CnB1 whereas previous studies were performed in the animals with relatively acute treatment of calcineurin inhibitors (1, 2, 9, 32). Considering the physiological significance for maintaining a proper renal E_K_ and K^+^ homeostasis, it is conceivable that factors other than PP2B may be able to compensate the function of calcineurin of the DCT through a tubule-remodeling process.

**Figure 8:**
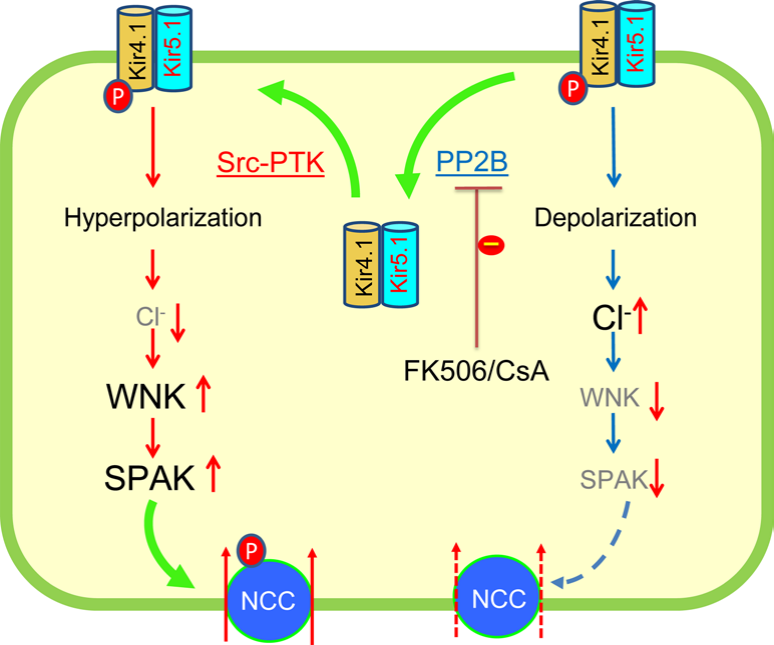
A cell scheme illustrating the role of PP2B in regulating the basolateral Kir4.1/Kir5.1 in the DCT. The large and bold font size represents the stimulation whereas grayed and small font size represents the inhibition. Src-PTK, Src family protein tyrosine kinase; WNK, with-no-lysine kinase; SPAK, ste20 proline and alanine-rich protein kinase.

The finding that acute inhibition of PP2B stimulates the basolateral Kir4.1/Kir5.1 of the DCT suggests that calcineurin may be involved in determining the baseline activity of Kir4.1/Kir5.1. Figure 8 is a scheme illustrating the role of PP2B in regulating the basolateral Kir4.1/Kir5.1 activity in the DCT. We speculate that PP2B may play a role in fast stimulation of renal E_K_ by targeting Kir4.1/Kir5.1 in response to an acute K^+^ loading because the basolateral Kir4.1/Kir5.1 in the DCT is a key component of the “K-sensor” mechanism (11, 14–16, 24). Since Kir4.1/Kir5.1 is also a major type of basolateral K^+^ channel in the CCD (43–45), the inhibition of calcineurin with FK506 or CsA is expected to hyperpolarize the membrane potential of the CCD thereby increasing the driving force of Na^+^ absorption through ENaC. Thus, it is possible that FK506 or CsA-induced increase in renal Na^+^ absorption is due to not only stimulating NCC activity but also enhancing ENaC function in the CCD. Accordingly, the selective inhibition of Kir4.1/Kir5.1 in the kidney would be a potential approach to treat CsA- or FK506-induced hypertension. We conclude that the acute inhibition of calcineurin stimulates the basolateral Kir4.1/Kir5.1 activity of the DCT, and CsA- or FK506-induced stimulation of Kir4.1/Kir5.1 is, at least in part, contributes to the calcineurin inhibition–induced stimulation of NCC.

## Methods

All supporting data and detailed methods including animal preparation, electrophysiology, Western blot, fluorescence immunostaining, and in vivo measurement of HCTZ-induced natriuresis are available within the article and its online supplement.

### Generating KS-Kir4.1–KO and Ks-FKBP12–KO mice.

Mice expressing Pax8-rtTA and tet-on LC-1 transgene (both from our lab, New York Medical College) were crossed with *Kcnj10^fl/fl^* or *Fkbp1a^fl/fl^* mice (both from David H. Ellison, Department of Medicine, Oregon Health & Science University, Portland, Oregon, USA) to generate inducible kidney tubule–specific Kir4.1-KO (Ks-Kir4.1–KO) and Ks-FKBP12–KO mice, respectively. *Kcnj10* or *Fkbp1a* deletion was conducted in 8-week-old male and female mice homozygous for floxed *Kcnj10* or *Fkbp1a* gene and heterozygous for Pax8-rtTA/LC-1 transgene by providing doxycycline (5 mg/mL, 5% sucrose) in the drinking water for 2 weeks. This was followed by at least 2 additional weeks without doxycycline treatment before performing experiments. Littermate mice of the same age and genetic background drinking 5% sucrose were used as controls (*Kcnj10^fl/fl^* or *Fkbp1a^fl/fl^*). Tail DNA was PCR-amplified and the primers for genotyping are shown in Supplemental Table 1.

### Preparation of the DCT.

Mice were sacrificed by CO_2_ inhalation plus cervical dislocation. The abdomen of the mice was quickly opened to expose the left kidney, which was then perfused with 2 mL L-15 medium (Life Technologies) containing type 2 collagenase (250 U/mL). After the perfusion, the left kidney was removed for harvesting the renal cortex, which was further cut into small pieces and incubated in collagenase-containing L-15 media for 30–50 minutes at 37°C. The tissue was then washed 3 times with fresh L-15 medium and transferred to an ice-cold chamber for dissection. The isolated DCTs were placed on a small cover glass coated with poly-lysine, and the cover glass was placed on a chamber mounted on an inverted microscope (TE300, Nikon Japan).

### Patch-clamp experiments.

A Narishige electrode puller was used to make the patch-clamp pipettes from Borosilicate glass (1.7 mm outer diameter). The resistance of the pipette was 5 MΩ (for single-channel recording) or 2 MΩ (for whole-cell recording) when it was filled with solution containing (in mmol/L) 140 KCl, 1.8 MgCl_2_, and 10 HEPES (titrated with KOH to pH 7.4). We have used the single-channel recording to examine Kir4.1/Kir5.1 channel activity, defined as NP_o_, and used the perforated whole-cell recording to measure Ba^2+^-sensitive Kir4.1/Kir5.1-mediated K^+^ currents and [I] reversal potential in the isolated DCT. The detailed method for the patch-clamp experiment is described in Supplemental Methods.

### Immunoblotting.

Whole kidney protein extract was obtained from frozen kidney homogenized in a buffer containing 250 mM sucrose, 50 mM Tris-HCl (pH 7.5), 1 mM EDTA, 1 mM EGTA, and 1 mM DTT supplemented with phosphatase and protease inhibitor cocktails (Sigma-Aldrich). Protein (40–60 μg) was separated on 4%–12% (wt/vol) Tris-glycine gel (Novex, Thermo Fisher Scientific) and transferred to a nitrocellulose membrane. The membranes were incubated 1 hour with LI-COR blocking buffer (PBS) and then incubated overnight at 4°C with anti-NCC (1:2,000; AB3553), anti-pNCC at threonine 53 (1:2,000; THr53), and anti-Kir4.1 antibodies (1:1,000; APC-035-AG); see *Materials* for manufacturers. All antibodies used in the experiments were validated previously (46, 47). An Odyssey infrared imaging system (LI-COR) was used to capture the images at a wavelength of 680 or 800 nm.

### Perfusion fixation and tissue processing.

Adult (10–12 weeks) *Kcnj10^fl/fl^* (control) and Ks-Kir4.1–KO mice (*n* = 4) were anesthetized with isoflurane and pentobarbital sodium and perfused via the abdominal aorta with PBS for 30 seconds followed by 3% paraformaldehyde/PBS for 5 minutes to fix the kidneys. Kidney tissue was proceeded for paraffin-embedding and sectioning (4 μm). Kidney sections were dewaxed and boiled in citrate buffer (pH 6) for 5 minutes for antigen retrieval followed by incubation with blocking medium (5% skim milk/PBS for 30 minutes). For double labeling of Kir4.1 and pNCC (pS71-NCC), the antibodies against Kir4.1 (guinea pig anti-Kir4.1; Alomone Labs) and pNCC (rabbit anti–pS71-NCC, generated in-house) (1) were sequentially applied for 1 hour, separated by a washing step. Fluorescent Cy2- or Cy3-conjugated antibodies (705225147 and 111-165-205, Dianova, BIOZOL) were used for detection. The signal was evaluated in a Leica DMRB or a Zeiss confocal microscope (LSM 5 Exciter). Kir4.1 fluorescence signal was semiquantified using ZEN and ImageJ software (NIH) as described previously (1).

### In vivo measurement of HCTZ-induced natriuresis.

Animals were anesthetized by peritoneal injection of inactin (100 mg/kg BW). The mice were placed on a heated small blanket to maintain body temperature at 37°C. The trachea was cannulated to clear any mucus that may be produced during the experiment. A carotid artery was catheterized with PE10 tubing for blood collection; jugular vein was also cannulated for i.v. infusion. The bladder was exposed and catheterized via a suprapubic incision with a 10 cm piece of PE10 tubing for urine collections. After completion of surgery, isotonic saline was given i.v. for 4 hours (0.25–0.3 mL/1 h and total 1.0–1.2 mL 0.9% saline) to replace surgical fluid losses and to maintain hemodynamics. Urine collections started 1 hour after infusion of 0.3 mL saline, and 6 collections (every 30 minutes) were performed (2 for controls and 4 for experiments). After 6 urine collections, the mice were sacrificed by i.v. somnasol.

### Materials.

Chemicals including inactin, CsA, PP1, and HCTZ were purchased from Sigma-Aldrich whereas tacrolimus was obtained from Alfa Aesar. We purchased GAPDH antibody from Sigma-Aldrich, NCC antibody from MilliporeSigma, pNCC (Thr53) antibody from PhosphoSolutions, and Kir4.1 antibody from Alomone Labs. Antibody for pNCC (Ser71) was developed in-house.

### Statistics.

We used software (Sigma plot 14) for the statistical analysis. For analyzing the values between 2 groups we used 1-tailed *t* test, and for comparisons of the values within the same group, we used paired 1-tailed *t* test. We used 1-way or 2-way ANOVA for analyzing results of more than 2 groups, and Holm-Šídák test was used as post hoc analysis. *P* values less than 0.05 were considered statistically significant. Data are presented as the mean ± SEM.

### Study approval.

New York Medical College’s independent IACUC has approved the animal care and animal use (IACUC number 69-2-1120). 

## Author contributions

DDZ, XPD, KM, FR, YX, JYZ, and DHL conducted the experiments and analyzed data. DDZ, XPD, DHL, and WHW designed the study. DDZ, XPD, KM, DHL, and WHW drafted the manuscript.

## Supplementary Material

Supplemental data

## Version 1 02/23/2023

In-Press Preview

## Version 2 04/10/2023

Electronic publication
