# Supplementary material for: Calcineurin inhibitors stimulate Kir4.1/Kir5.1 of the distal convoluted tubule to increase NaCl cotransporter
Source: JCI Insight. 2023 Apr 10;8(7):e165987. doi: 10.1172/jci.insight.165987 (PMC10132170; doi:10.1172/jci.insight.165987)
Supplement: Supplemental data [file jciinsight-8-165987-s109.pdf]

Supplementary material

"Calcineurin-inhibitors stimulate Kir4.1/Kir5.1 of distal-convoluted-tubule to increase Na-Cl cotransporter (NCC) "

Methods for single channel recording and whole-cell recording

Single-channel recording An Axon 200B patch-clamp amplifier was used to record the single K^+ channel currents, which were low-pass filtered at 1 KHz and digitized by an Axon interface (Digidata 1332). Channel activity, defined as NP_o (a product of channel number and open probability), was calculated from data samples of 60 seconds duration in the steady state as follows:

$$NP_o = \sum (1t_1 + 2t_2 + \dots it_i),$$

where t_i is the fractional open time spent at each of the observed current levels. The channel conductance was determined by measuring the current amplitudes over several voltages. The bath solution contains (in mM) 140 NaCl, 5 KCl, 1.8 $CaCl_2$, 1.8 $MgCl_2$, and 10 HEPES (pH 7.4) and the pipette solution contains (in mM) 140 KCl, 2 $MgCl_2$, 1 EGTA and 5 HEPES (pH 7.4). Data were analyzed using the pCLAMP Software System 9 (Axon).

Whole-cell recording An Axon 200A patch-clamp amplifier was used to record the whole-cell K^+ currents, which were low-pass filtered at 1 KHz, digitized by an Axon interface (Digidata 1440A). Data were analyzed using the pCLAMP Software System 9 (Axon). The pipette solution was the same as that for the single channel recording but with 0.5 mM MgATP, whereas the bath solution contains (in mM) 140 KCl, 1.8 $CaCl_2$, 1.8 $MgCl_2$, and 10 HEPES (pH 7.4). The whole-cell K^+ current was determined by adding 1 mM Ba^{2+} in the bath solution. For measuring the reversal potential of inward-to-outward current ([I]-reversal-potential) using perforated whole-cell recording, the isolated DCTs were super-fused with

HEPES-buffered 140 mM NaCl solution. The pipette was filled with 140 mM KCl pipette solution and was then backfilled with amphotericin B (20 mg/0.1ml). After forming a high-resistance seal (>2 GΩ), the membrane capacitance was monitored until the whole-cell patch configuration was formed. The voltage at which the inward K^+ currents were zero was the I_K reversal potential determined by a ramp protocol from -100 to 100 mV.

Table s1 Primers for genotyping

***Kcnj10* flox**	Forward 5'-TGATGTATCTCGATTGCTGC-3'<br>Reverse 5'-CCCTACTCAATGCTCTTAAC-3'	Flox 550 bp<br>WT 420 bp
***Fkbp1a* Flox**	Forward 5' AGAACTTGCCCTTCAGTATT-3'<br>Reverse 5' AGGCTTGTACCACTATTTTCT-3'	Flox ~590 bp<br>WT ~700 bp
**Pax8rtTA**	Forward 5' CCATGTCTAGACTGGACAAGA-3'<br>Reverse 5'-CAGAAAGTCTTGCCATGACT-3'	220 bp
**LC1-CRE**	Forward 5'-TTTCCCGCAGAACCTGAAGATG-3'<br>Reverse 5'-TCACCGGCATCAACGTTTTCTT-3'	190 bp

Results

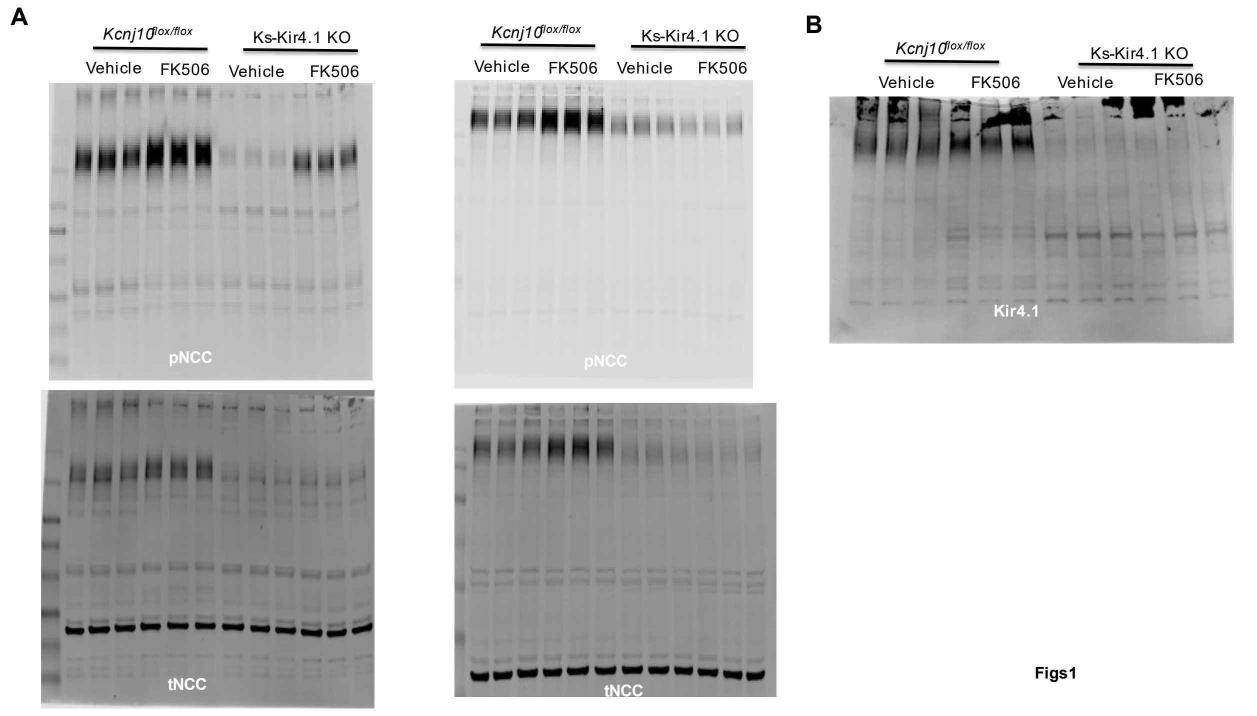

Fig.1s FK506 treatment-induced stimulation of pNCC expression is attenuated in Ks-Kir4.1 KO mice. **(A)** Two western blots show the abundance of $pNCC^{T53}$ and tNCC in male *Kcnj10$^{flox/flox}$* mice and in Ks-Kir4.1 KO mice treated with vehicle (control) and FK506 (0.75 mg/Kg BW). FK506 or vehicle was applied by peritoneal injection 30 min before the experiment. (B) A Western blot shows the expression of Kir4.1 in the control and Ks-Kir4.1 KO mice to validate the deletion of Kir4.1. Kir4.1 band represents a Kir4.1 homotetramer or Kir4.1/Kir5.1 heterotetramer. For the western blot experiments, the lysate obtained from the renal cortex tissue was unheated.

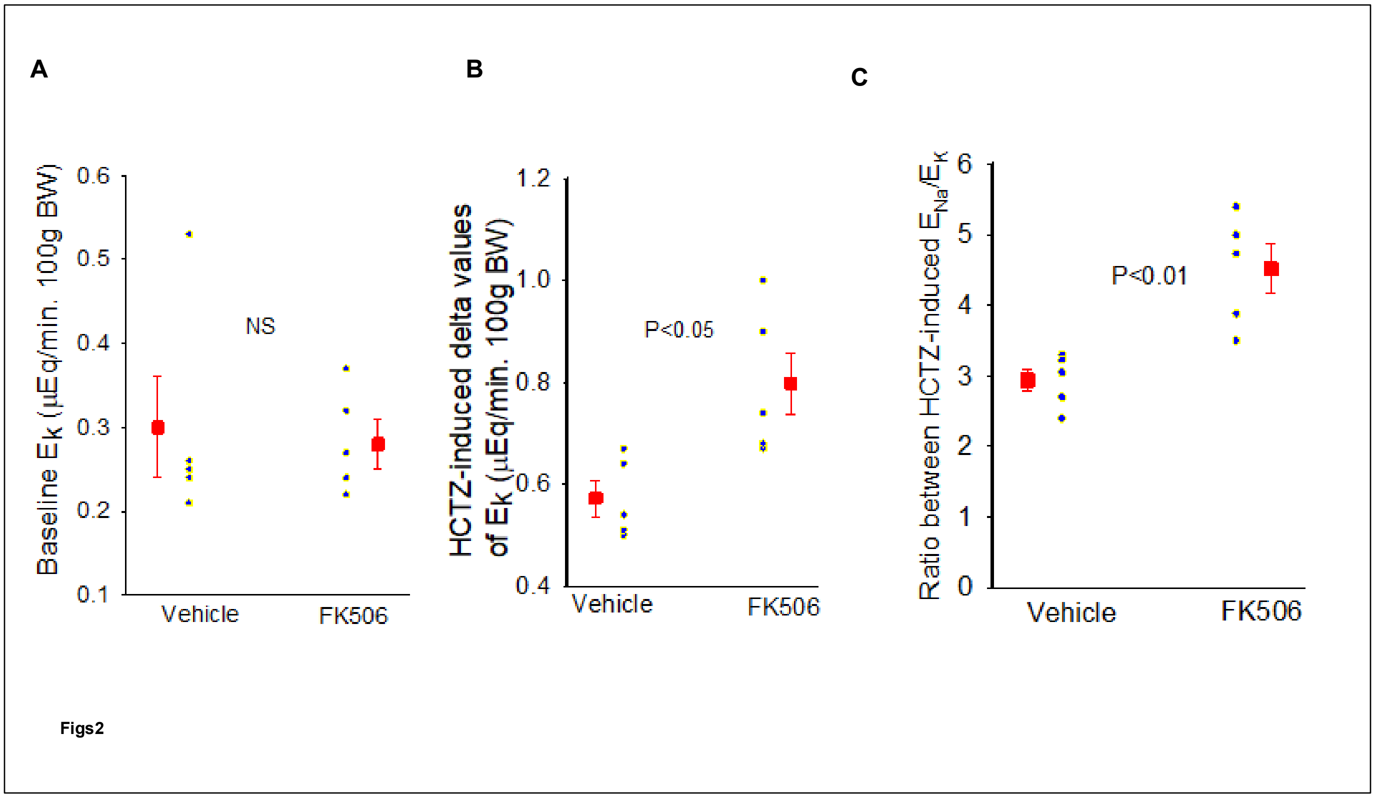

Fig.s2 A scatter plot shows the mean value (red square) and each single data point of the baseline renal K^+ excretion (E_K) (A) , HCTZ (30 mg/kg BW) -induced net E_K (B), and the ratio between HCTZ-induced E_{Na} and E_K (C) in the male control mice (*Kcnj10*$^{flox/flox}$ and *Fkbp1a*$^{flox/flox}$) treated with vehicle or FK506 (0.75 mg/Kg BW) injection for 30min before the experiments. The baseline E_K was measured after the mice received initial saline perfusion (0.3 ml one hour before measurement). The significance is determined by unpaired *t* test.

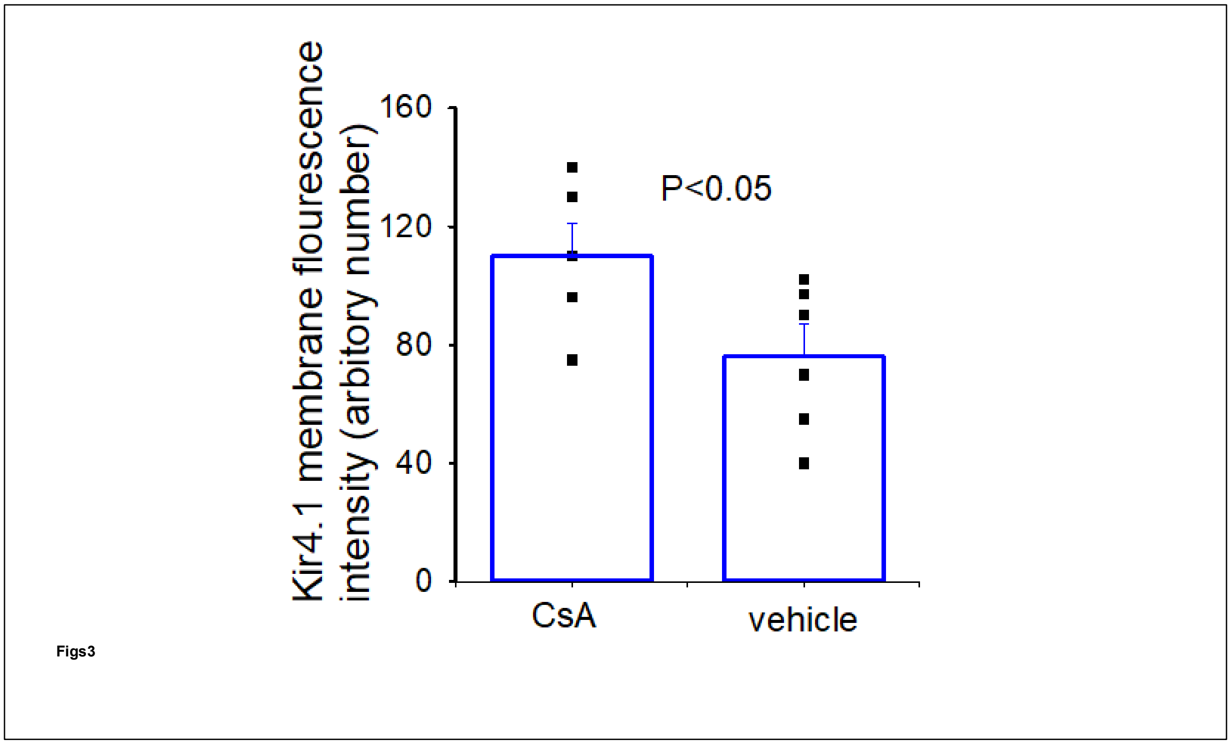

Fig.s3 A bar graph with scatter plot shows relative intensity of Kir4.1 fluorescence signaling at the basolateral membrane of the DCT in the mice treated with vehicle or CsA (3 mg/Kg BW). CsA or vehicle was applied by peritoneal injection 30 min before the experiment. The signal was evaluated using ZEN and ImageJ software. The significance is determined by unpaired *t* test.
